# *In vivo* photoacoustic imaging dynamically monitors the structural and functional changes of ischemic stroke at a very early stage

**DOI:** 10.7150/thno.38554

**Published:** 2020-01-01

**Authors:** Jing Lv, Shi Li, Jinde Zhang, Fei Duan, Zhiyou Wu, Ronghe Chen, Maomao Chen, Shanshan Huang, Haosong Ma, Liming Nie

**Affiliations:** State Key Laboratory of Molecular Vaccinology and Molecular Diagnosis & Center for Molecular Imaging and Translational Medicine, School of Public Health, Xiamen University, Xiamen, China.

**Keywords:** ischemic stroke, photoacoustic imaging, early diagnosis.

## Abstract

Ischemic stroke (IS) is one of the leading causes of death and accounts for 85% of stroke cases. Since the symptoms are not obvious, diagnosis of IS, particularly at an early stage, is a great challenge. Photoacoustic imaging combines high sensitivity of optical imaging and fine resolution of ultrasonography to non-invasively provide structural and functional information of IS.

**Methods**: We adopted three rapid photoacoustic imaging systems with varying characteristics, including a portable handheld photoacoustic system, high-sensitivity bowl-shaped array photoacoustic computed tomography (PACT), and high-resolution photoacoustic microscopy (PAM) to assess the stereoscopic and comprehensive pathophysiological status of IS at an early stage. Two representative models of IS, referring to photothrombosis and middle cerebral artery occlusion (MCAO) models, were established to verify the feasibility of photoacoustic imaging detection.

**Results:** Non-invasive, rapid PACT of the IS model in mouse provided structural information of the brain lesion, achieving early disease identification (5 min after the onset of disease). Moreover, it was able to dynamically reflect disease progression. Quantitative high-resolution PAM allowed observation of pathological changes in the microvascular system of mouse brain. In terms of functional imaging, significant differences in oxygen saturation (sO_2_) levels between infarcted and normal areas could be observed by PACT, permitting effective functional parameters for the diagnosis of IS.

**Conclusions:** We used PACT to perform full-view structural imaging and functional imaging of sO_2_ in IS at the macroscopic level, and then observed the microvascular changes in the infarcted area at the microscopic level by using PAM. This work may provide new tools for the early diagnosis of IS and its subsequent complications as well as assessment of disease progression.

## Introduction

Over the past two decades, stroke has emerged as the second most common cause of death worldwide 1, 2. Although many advances have been made in the diagnosis and treatment of stroke, the morbidity and mortality rates still remain unacceptably high 3. Stroke is composed of hemorrhagic stroke and ischemic stroke (IS), with IS accounting for 85% of cases 4. IS is caused by obstruction of brain vessel(s), resulting in brain neuronal death in a few seconds 5, 6. If diagnosis and treatment are not timely, IS can lead to persistent brain damage, long-term disability, and even death. Timely administration of thrombolytic therapy, such as recombinant tissue plasminogen activator within 3 hours after IS, is a direct and effective treatment 7. However, due to delayed diagnosis, only 2% of patients receive recanalization therapy to restore perfusion 8. Therefore, achieving early diagnosis of IS is of major importance for a favorable patient prognosis. In addition, some patients carry a risk of hemorrhagic transformation, characterized by potential disability and high mortality 9. Thus, it is also crucial to identify whether ischemic transformation accompanies the onset of IS in a timely and early manner. Moreover, the anatomical location of the ischemic lesion and functional imaging of cerebral oxygen saturation at the early stage of stroke might allow for timely therapeutic intervention 10, 11.

Although stroke is manifested in a variety of clinical syndromes, neuroimaging is the most important biomarker to help distinguish stroke subtypes and assess treatment eligibility 12. In the clinic, computed tomography (CT) and magnetic resonance imaging (MRI) are the two most commonly used imaging methods for the diagnosis of IS 13, 14. The development of CT, MRI, and other imaging technologies has greatly improved the survival rate of stroke patients. However, CT is ionizing and insensitive to IS, especially at the early stage of the disease. Further, T2-weighted imaging plays an important role in imaging of IS, but suffers from low sensitivity in early diagnosis 15, 16. Diffusion-weighted imaging can detect IS sensitively at the early stage; however, it is usually time consuming, with long imaging acquisition and processing times severely delaying treatment initiation, while IS is a medical emergency. Therefore, a non-invasive and expedite imaging technology with high resolution and sensitivity for accurate diagnosis of IS at an early stage is urgently required.

Photoacoustic imaging (PAI) is a new non-invasive and radiation-free imaging method that has rapidly developed in recent years. By acoustically detecting photons absorbed by tissue, PAI breaks the resolution and depth limitations of pure optical imaging, allowing the rich contrasts of optical imaging as well as the high-resolution of ultrasound (US) imaging at depths of several millimeters to centimeters in living tissues 17-20. PAI provides an important means to study morphological lesions, functional metabolism, and the physiological and pathological characteristics of biological tissues, along with long-term dynamic monitoring. It has demonstrated promising imaging capabilities, ranging from organelles to organs, and in particular for the structural imaging of cardiovasculature since this method is intrinsically sensitive to hemoglobin 21-23. Furthermore, PAI has created a new frontier in the bioimaging of brain connectivity and functional disorders 24, 25. Using endogenous contrasts, scientists have applied a functional photoacoustic microscopy (PAM) system to image the cerebral metabolism and vascular morphology of a mouse brain 26-28. The results showed that the technique possesses high sensitivity to blood monitoring and is capable of excellent functional imaging of hemodynamics. However, limited studies have reported on the application of this technique for IS imaging, let alone the precise localization of infarct site and monitoring of blood oxygen function.

Herein, we applied a versatile PAI technique aided with Evans blue (EB) dye to dynamically reflect the structural and functional characteristics of IS on mice at a very early stage, thus closing the gap between photoacoustic (PA) research and IS. Simplistically, the underlying mechanism involves the partial destruction of the blood-brain barrier (BBB) at the infarct site upon occurrence of IS. Thus, EB dye bound to the plasma albumin crossed the BBB and accumulated in the infarcted area, significantly enhancing the PA signal.

**Scheme 1:**
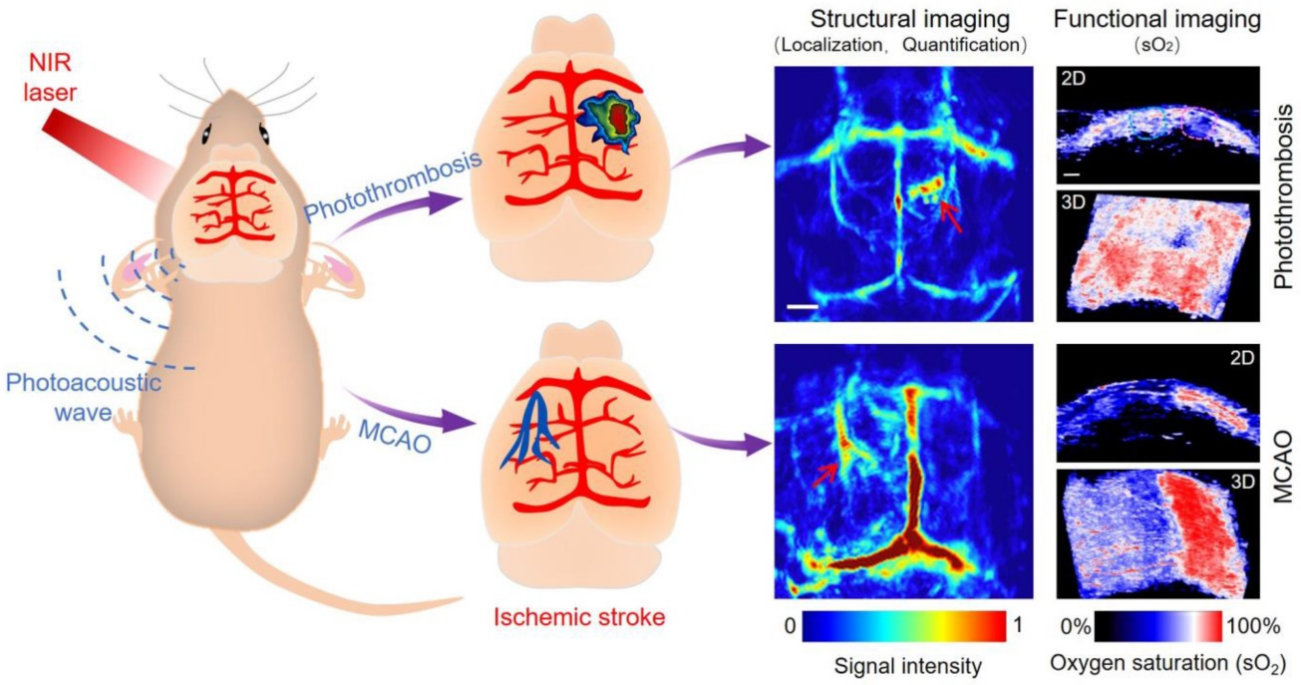
Schematic illustration of structural and functional photoacoustic imaging on photothrombosis/MCAO model mice at an early stage *in vivo.* Scale bar, 1 mm.

Two representative models of IS, referring to photothrombosis and middle cerebral artery occlusion (MCAO), were established to verify the feasibility of the technique 29-31. The occlusion of the middle cerebral artery is close to human IS and shows penumbra similar to human stroke. Because of the simplicity of surgical procedures and the controllability and high reproducibility of injury area, photothrombosis model is increasingly popular in the study of IS 32, 33. PAI was used to study the two models as schematically illustrated in Scheme 1. The experimental results indicated that this technique could directly image the infarct region in the photothrombosis model (5 min after IS). In the MCAO model, cerebral cortical venous enlargement could be imaged by photoacoustic computed tomography (PACT) even without the need for EB dye, indirectly reflecting the physiopathological features around the infarction area as early as 3 min following IS and allowing surveillance of the obstruction of cerebral cortical venous return.

Functionally, we clearly and dynamically observed significant changes of oxygen saturation (sO_2_) in infarcted areas in both models. The sO_2_ in the infarcted area decreased significantly compared with the normal area, further indicating the occurrence of IS. Moreover, PAI could also quickly and directly scrutinize the occurrence of hemorrhagic transformation, potentially guiding doctors to adopt a reasonable therapeutic regimen. In addition, the acquisition time was no longer than 3 min and 0.2 s per frame, thus effectively overcoming time-consuming data acquisition in other imaging techniques, saving valuable treatment time for the patients. Our study showed that PAI could provide a rapid method for the diagnosis of IS in mice and also expand related pre-clinical study range, as well as the progression mechanism study.

## Results

### PACT of photothrombosis model at an early stage *in vivo*

Firstly, the IS model was induced by photothrombosis on mice to investigate the pathological changes by PACT at 680 nm. The mice were then immediately injected with EB dye (2 mg/kg), which exhibited a broad absorption band from 500 to 700 nm with an absorption peak at 610 nm (Figure S1). PA images of EB dye at different concentrations were obtained by PAM at 532 nm (Figure S2) and PACT at 680 nm (Figure S3). The results suggested that EB could be used as an efficient PA contrast agent at 532/680 nm.

**Figure 1:**
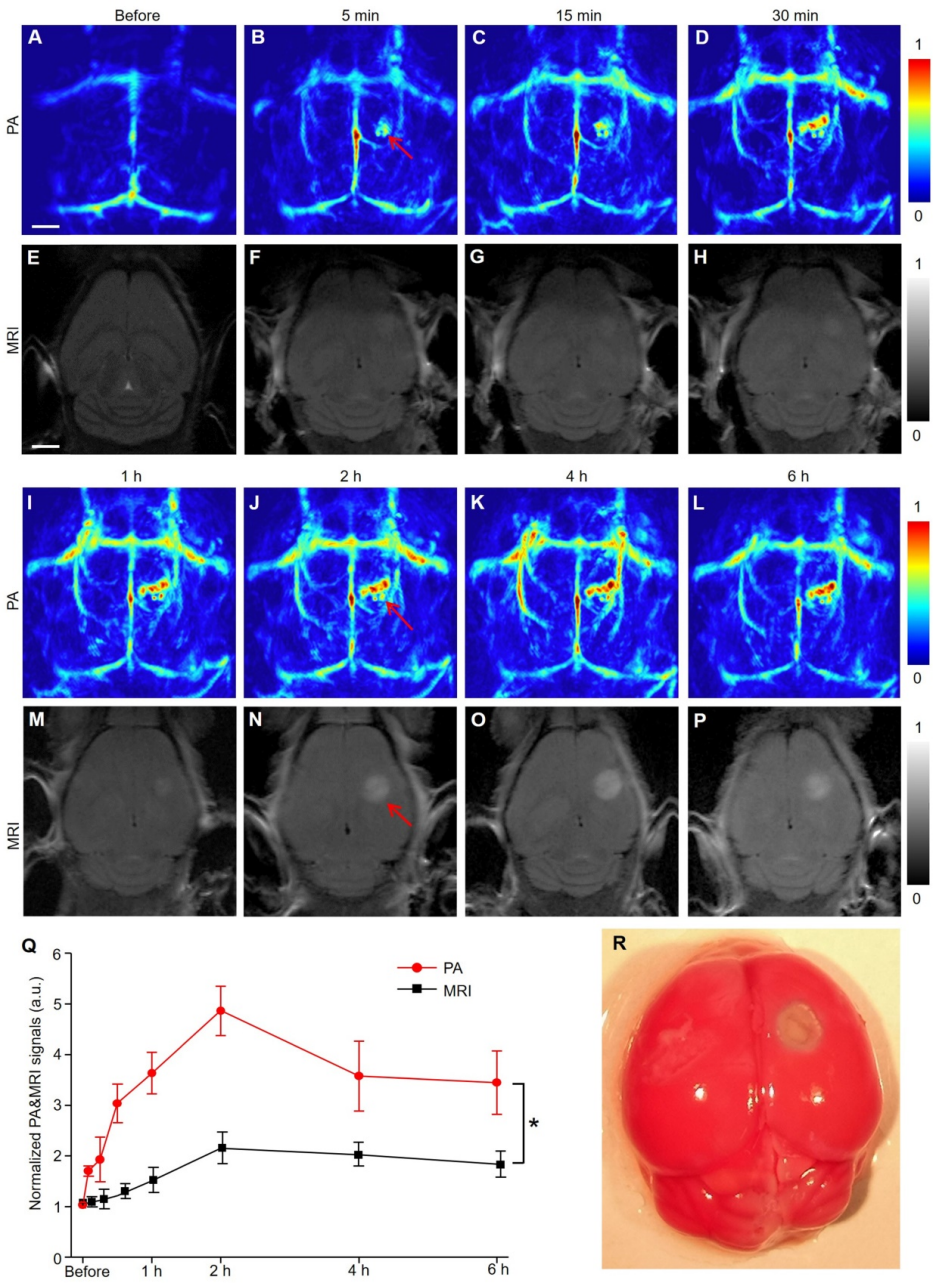
** PACT and MRI of mouse brain in a photothrombosis model at an early stage *in vivo*.** (**A-D**), (**I-L**) Representative PA images of a photothrombosis mouse model right followed by EB dye injection at varied time points upon injection at 680 nm. (**E-H**), (**M-P**) Representative MR images of a photothrombosis mouse model at varied time points. (**Q**) Normalized PAI and MRI signals of mice brains (*n* = 5; the error bars show the standard deviation) in infarcted areas at varied time points upon EB dye injection. (*)* P <* 0.05*.* (**R**) Triphenyl tetrazolium chloride staining in the brain of model mice. Scale bar, 1 mm.

PACT and MRI were simultaneously employed to detect the infarct area at different time points (Figure 1). Five minutes after EB dye injection, we observed an area with a significantly increased PA signal (indicated by a red arrow in Figure 1B). The area gradually expanded over time and formed a caterpillar-like shape (indicated by a red arrow in Figure 1J). The PA signal intensity in this region increased rapidly within 2 h and gradually decreased over time to reach a plateau (Figure 1Q). The viable tissue was stained red and the non-viable infarct tissue remained white following triphenyl tetrazolium chloride (TTC) staining of the mouse brain (Figure 1R and S4), showing excellent consistence with our PA observations. Furthermore, the microscopic images of brain tissue slices (H&E staining) revealed different pathological manifestations in both infarcted and non-infarcted areas. In the infarcted area, the numbers of nerve cells decreased and were irregularly located. Vacuoles formed in the cytoplasm with obvious interstitial enlargement and nuclear pyknosis (Figure S5). Therefore, we concluded that the noticeable PA signal changes indicated the infracted area.

PACT images of a mouse brain of IS are illustrated in Supplementary Figure S6. Several major blood vessels, including the superior sagittal sinus, transverse sinus, and other cortical veins, were clearly visualized (Figure S6A). In addition, the infarct was also clearly displayed, as marked in a white dotted circle.

Representative MR images at the corresponding time points after IS of mice model (Figure 1E-H, M-P) showed that the signal of the infarct location was almost negligible within the first hour and difficult to identify until after 2 hours. By quantifying the signal value of the infarct area, PACT had a much stronger signal-to-background ratio (approximately twofold) than the MR image in Figure 1Q (*P* < 0.05). The time of data acquisition was 1.7 min for PAI in contrast to 5 min for MRI. In summary, PACT allowed the dynamic observation of the infarct area with the prolongation of ischemic time. Furthermore, PACT could detect the infarct area at a much earlier stage than conventional MRI in this study (5 min versus 2 h) with a faster imaging time (1.7 min versus 5 min).

### PACT of MCAO model at early stage *in vivo*

**Figure 2:**
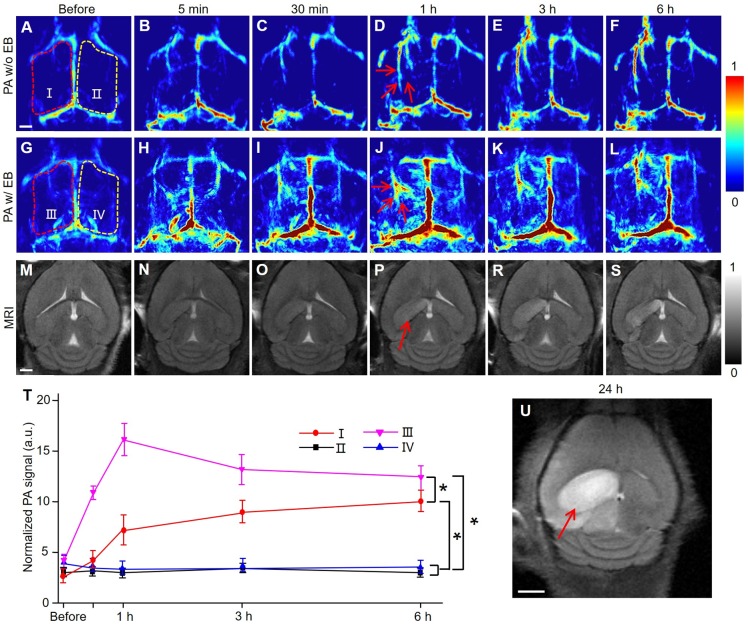
** PACT and MRI of mouse brain in MCAO model at an early stage *in vivo*.** (**A-F**) Representative PA images of an MCAO model mouse at varied time points without EB dye at 680 nm. (**G-L**) Representative PA images of an MCAO model mouse right followed by EB dye injection at varied time points upon injection at 680 nm. (**M-S**) Representative MR images of an MCAO model mouse at varied time points. (**T**) Normalized PA signals of mice brains (*n* = 5; the error bars show standard deviation) in the region of interest (regions I, II, III, IV) at varied time points (*)* P*<0.05. (**U**) MR image in the brain of a MCAO model mouse at 24 h. Scale bar, 1 mm.

Similarly, the IS model was established by the MCAO method on the left hemispherical brain of mice to investigate the pathological changes by PACT at 680 nm. Regions I and III refer to the hemisphere of IS, while regions II and IV refer to the normal hemisphere (Figure 2A-I). The top panel (Figure 2A-F) represented PA images of a mouse brain at different time points without injection of EB dye. PAI of region I showed that the blood signals in the cerebral cortex of mice increased gradually with the progress of IS. Upon EB dye injection (2 mg/kg), hemispheric PA signals in the IS area were enhanced significantly (region III in Figure 2G-L). Conversely, in regions II and IV where IS did not occur, PA signals remained practically unchanged within 6 hours (*P* > 0.05). The blood signals in regions I and III were more significantly enhanced than those in regions II and IV (*P* < 0.05). The changes in blood signal in region III were more significant than those in region I because of EB dye injection (*P* < 0.05) (Figure 2T). We infer that the PA signal of blood in regions II and IV was due to the obstruction of cerebral venous reflux. After IS, brain edema occurred at the infarct sites 34, 35, as confirmed by MRI data (Figure 2U). The MR image in supplementary Figure S7 indicated that the infarcted area was 2 mm deep from the cerebral cortex. The occurrence of brain edema further led to the increase of intracranial pressure, causing the obstruction of venous return. TTC staining of the mouse brain also confirmed the successful establishment of the MCAO model and the infarct area was shown in the black arrow in Figure S8.

The photograph of the modeling mouse in Supplementary Figure S9 showed that there was evident venous engorgement as indicated by blue arrows in the infarcted area relative to the non-infarcted area. In addition to the enhancement of PA signals of blood, other signals could be found in this area. We speculated that these signals might be due to the extravasation of EB dye caused by BBB damage in the brain after IS since EB dye has a high affinity to plasma albumin in blood and is often used to detect the openness of the BBB 36, 37.

To further verify that these signals were caused by BBB damage, EB dye was injected into normal and IS model mice via the caudal vein (Figure S10). The results showed that there were no extra signals in the brain of normal mice except for the enhancement of the main blood vessel signals (Figure S10A-D). In the brain of model mice, bright PA signals emerged in lesion areas (red arrows in Figure S10E-H). The corresponding MRI results after IS on mice (Figure 2M-S) showed high-intensity T2 signals in the left hemisphere of the brain with IS 1 hour after modeling (red arrow in Figure 2P).

### PACT of hemorrhagic transformation model in ischemic stroke at an early stage *in vivo*

**Figure 3:**
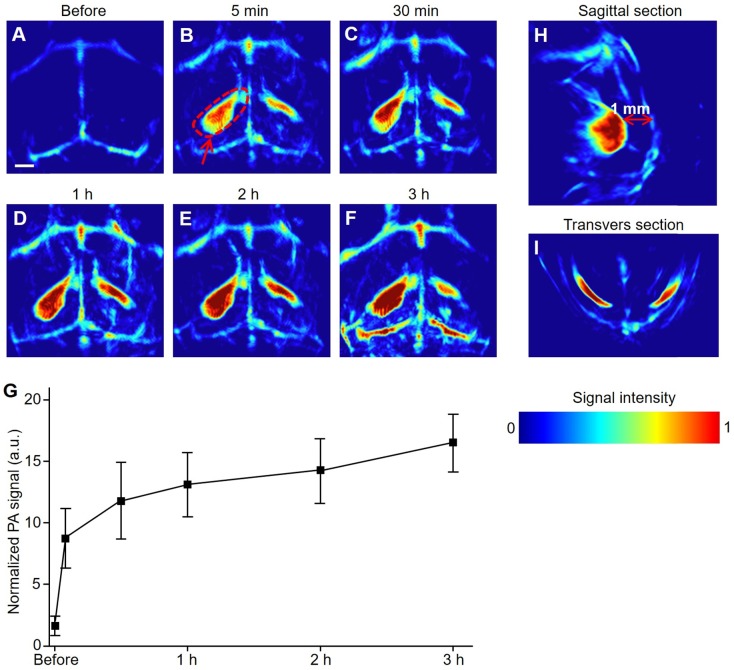
** PACT of mouse brain in a hemorrhagic transformation model at an early stage *in vivo*.** (**A-F**) Representative PA images of a hemorrhage transformation model mouse at varied time points at 800 nm. (**G**) Normalized PA signals of mice brains (*n* = 5; error bars show the standard deviation) in a hemorrhagic region at varied time points. (**H, I**) PA images from sagittal and transverse planes, respectively, of a model mouse brain. Scale bar, 1 mm.

The hemorrhagic transformation model was induced on mice brains to investigate the pathological changes by PACT at 800 nm. Except for several major blood vessels in the brain, no other significant PA signals were observed before modeling (Figure 3A). In particular, once the hemorrhagic foci were formed, a remarkable increase in PA signals was immediately observed (red arrows in Figure 3B-F). Furthermore, we observed that the PA signal intensity of the hemorrhagic focus on the left side was significantly higher than that on the right side, which was confirmed by H&E staining of mouse brain where hemorrhagic foci were indicated by black arrows (Figure S11).

Three hours after the modeling, the signal-to-background ratio at the hemorrhagic focus in the left (circled by red dotted lines) reached as high as 6:1. As illustrated in the transverse and sagittal sections (Figure 3H, I), the hemorrhagic focus was clear, not in the cerebral cortex but rather at a depth of 1 mm from the cerebral cortex. These results proved that PACT can non-invasively detect hemorrhagic foci at a very early stage (5 min). Simultaneously, the results could also promptly and intuitively indicate whether there is hemorrhagic stroke accompanied by IS.

### PAM of photothrombosis/MCAO model *in vivo*

**Figure 4:**
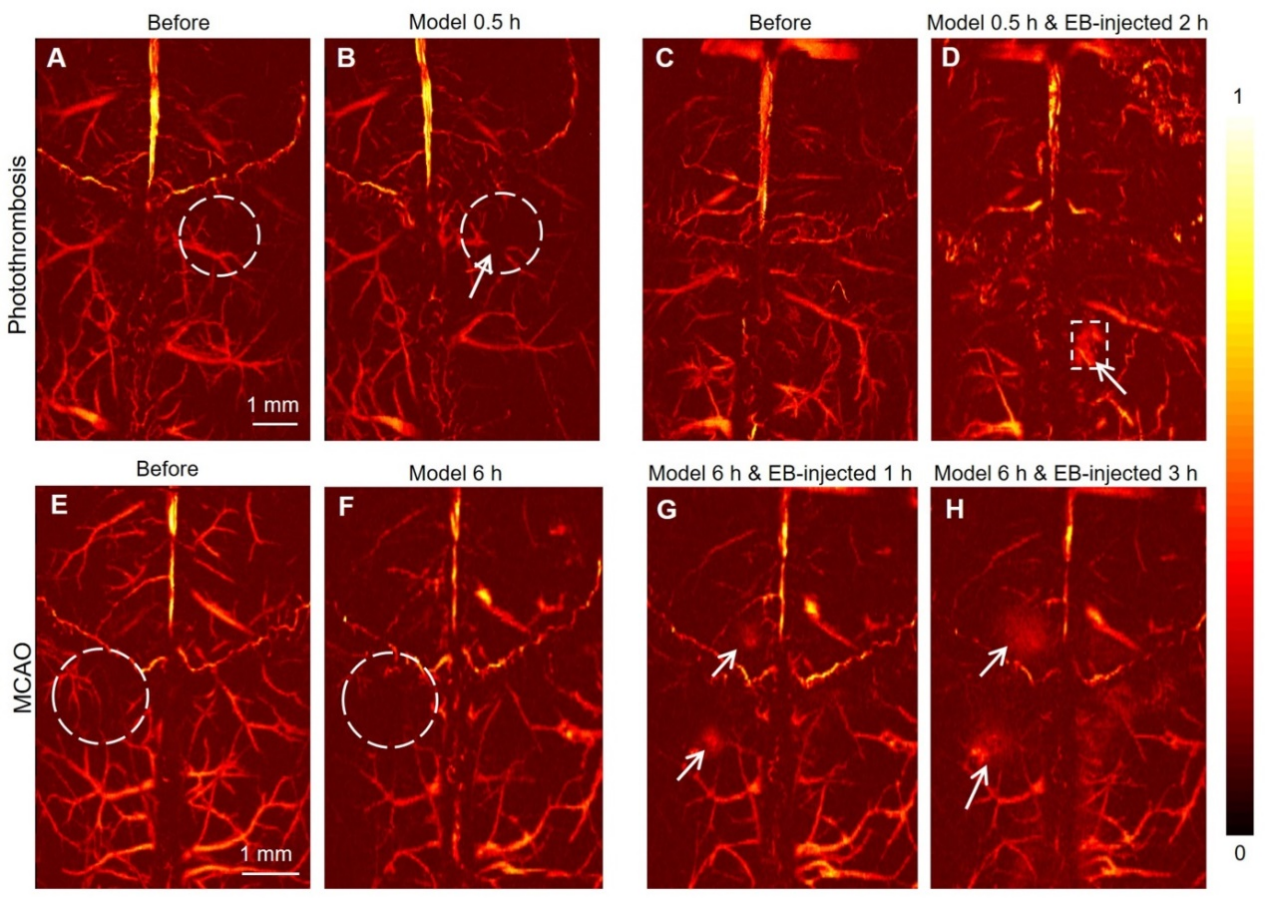
** PAM of mouse brain in photothrombosis and MCAO model *in vivo*.** (**A, B**) Representative PAM images of a mouse brain before and 30 min after photothrombosis modeling at 532 nm. (**C, D**) Representative PAM images of another mouse brain before and 2 h after photothrombosis modeling with EB dye injection, respectively. (**E, F**) Representative PAM images of a mouse brain before and after MCAO modeling after 6 h at 532 nm. (**G, H**) Representative PAM images of a mouse brain after MCAO modeling at 532 nm following EB injection after 1 h and 3 h, respectively.

PAM images of mice brains before and 30 min after the photochemistry stroke model at 532 nm (Figure 4A-B, respectively) showed that, besides large blood vessels, more subtle blood vessels could be clearly observed in the cerebral cortex of mice. Following laser irradiation, Rose Red B, a photosensitizer injected through the tail vein, was activated and reactive oxygen species were released, resulting in peroxidation injury of vascular endothelium. Subsequently, a thrombus was formed, which embolized the blood vessels leading to focal ischemic injury. Following photochemical thrombosis formation, the blood vessel (white arrow in Figure 4B) was obviously broken due to obstruction, demonstrating the mechanism of photothrombosis formation from a microscopic level 38, 39. On another mouse, photothrombosis model was established and 0.5 h later followed by EB injection (10 mg/kg) through the tail vein. PAM images of the cerebral cortex of the mouse before and 2 h after the EB injection (Figure 4C-D, respectively) clearly show the infarcted area (white dotted box in Figure 4D).

The PAM images of the cerebral cortex of mice before and 6 hours after MCAO modeling showed that the blood signals decreased significantly due to IS (Figure 4E-F, white dotted circle). EB dye was injected 6 h later after modeling, the exudation of EB in the cerebral cortex was remarkably observed (white arrows in Figure 4G, H). We speculated that the exudation of EB is due to the destruction of the BBB caused by IS, which would also confirm that the occurrence of IS could lead to the destruction of the BBB. Compared with PACT, PAM cannot only be used for the diagnosis of IS, but also offers better resolution and the visibility of a more subtle vascular structure in the brain of mice.

### Oxygen saturation mapping of mice brains before and after induction of ischemic stroke *in vivo*

**Figure 5:**
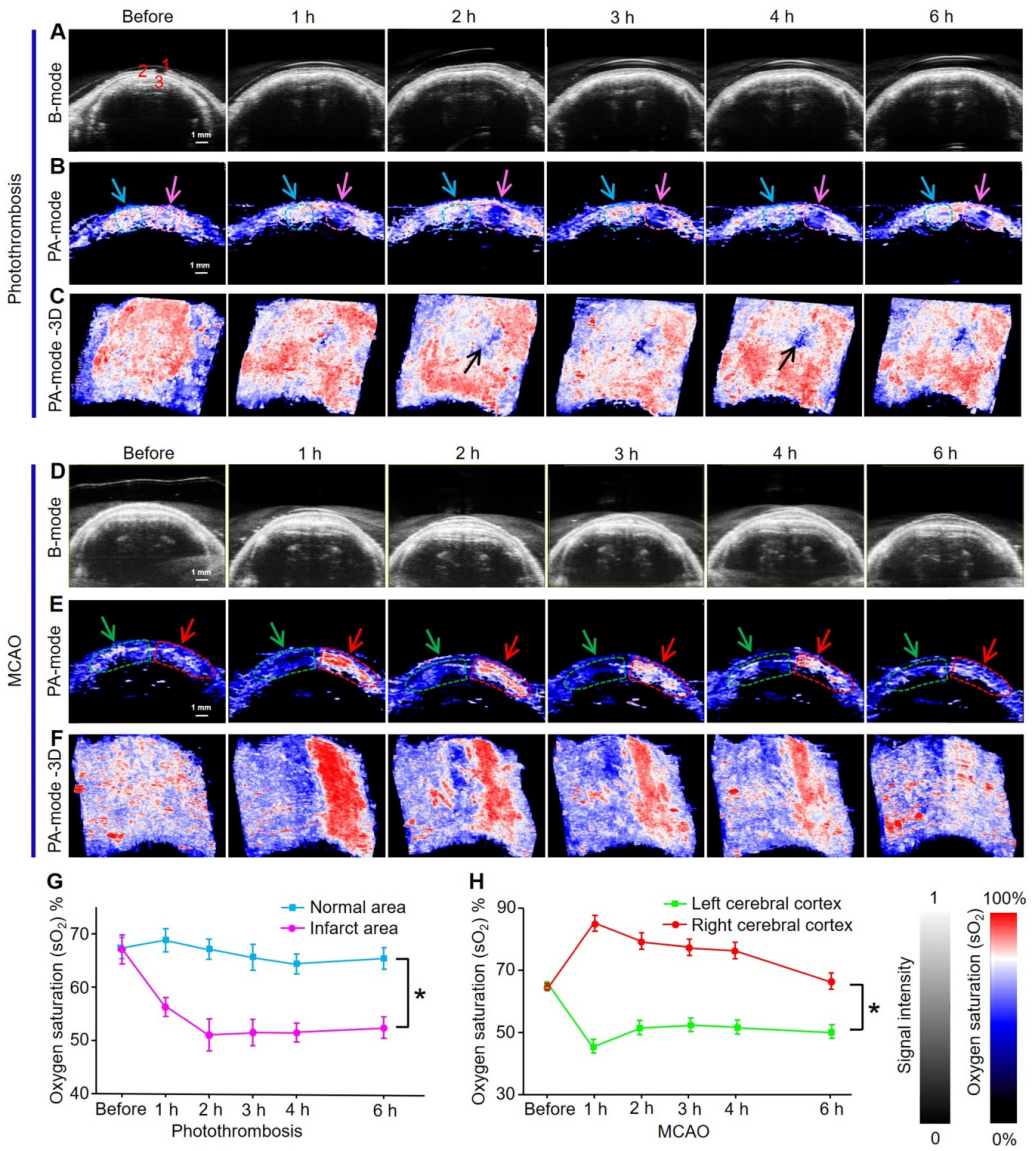
** Oxygen saturation mapping of mouse brain of IS using PACT *in vivo*.** (**A-C**) Ultrasonograms and 2D and 3D PA functional images of brain sO_2_ of photothrombosis model mice at different time points, respectively. Organ labeling part a (1) scalp, (2) skull, and (3) cerebral cortex. (**D-F**) Ultrasonograms and 2D and 3D PA functional images of brain sO_2_ of MCAO model mice at different time points, respectively. (**G, H**) Statistical results of sO_2_ mapping in apoplectic and normal areas at different time points of photothrombotic mice and MCAO mice (*n* = 5; the error bars show standard deviation), respectively. (*) *P <* 0.05.

PAI/US functional imaging of sO_2_ in mice brains at 750 and 850 nm produced two-dimensional (2D) and three-dimensional (3D) PA functional images and US images of the photothrombosis model at different time points (Figs 5A-C). The US images showed the scalp, skull, and cerebral cortex of mice as an anatomical reference (Figure 5A). The 2D pseudo-color PA sO_2_ images in the infarcted area were shown via spectral unmixing (Figure 5B); the red color represents a higher sO_2_ level while the blue represents a lower sO_2_ level. The PAI/US system records multiple 2D tomographic images and renders them in 3D. These 3D images showed the infarct area (black arrow Figure 5C) as blue, indicating a decrease in sO_2_ from 68% to 50% over time; while sO_2_ levels were maintained at approximately 65% in the normal area (Figure 5G). Quantitative analysis showed that sO_2_ in the infarcted area was significantly lower than that in the normal area (*P* < 0.05).

Because the cerebral infarction area in photothrombosis mice was relatively small, the mice have a longer survival time than MCAO mice. Therefore, we also monitored sO_2_ levels in the cerebral cortex of the model mice within 7 days (Figure S12A-C). Two identical region of interest (ROI) in each image were indicated by dashed circles; the green curve represents signals from the left cerebral cortex while the red curve exhibits signal from the right side where infarction occurs. The results illustrated that sO_2_ in the ipsilateral cortex of stroke mice dropped sharply from 75% to 38%, 37%, 35%, and 30% after 1 h and 3, 5, and 7 days after stroke, respectively (Figure S12D). sO_2_ levels on the left cerebral cortex increased notably from 75% to 85% at 1 h and 3 days after stroke and then returned to normal levels. The results suggested that the onset of stroke may continue to deteriorate the oxygenation of the ipsilateral cortex. PAI was able to directly and rapidly obtain this functional parameter, permitting long-term physiological status monitoring and prognosis prediction.

Analogously, US imaging and 2D and 3D PA functional images of brain sO_2_ levels in MCAO model mice at different time points were also assessed (Figure 5D-F). The pseudo-color images in the left hemisphere where IS occurred were blue, while in the right hemisphere red was observed (Figure 5F), indicating that the sO_2_ levels in the infarcted area decreased significantly within 6 hours in the left brain of mice. Compared with the photothrombosis model, the MCAO model had a much larger damage area and more serious damage to brain tissue. Quantitative results showed that sO_2_ levels dropped rapidly from 68% to 48% after 1 h, and then stabilized at approximately 50% in the left hemisphere. sO_2_ levels in the right hemisphere increased from 68% to 87% at 1 h and then slowly decreased to 68% (Figure 5H).

Quantitative analysis showed that the changes in sO_2_ levels in the left and right hemispheres were statistically different with time (*P* < 0.05). We speculate that the increase in sO_2_ levels in the right hemisphere was due to oxygen compensation. The significant decrease in sO_2_ levels in infarcted areas can be attributed to impaired brain function.

Functional PA imaging results indicated that sO_2_ levels in apoplectic and normal areas could be used as an effective parameter for the diagnosis of IS at an early stage. Furthermore, we found that the changes of sO_2_ were significantly different between the two stroke models. Because the infarct area of photothrombosis model was more localized, the sO_2_ of blood only decreased significantly in the infarct area. In the MCAO model, the whole sO_2_ of the hemisphere where the blood vessels were blocked decreased significantly. The results showed that the damage range of MCAO model was larger than that of photothrombosis model.

## Materials and methods

### Animal preparation

This study conforms to the standards of nursing and using laboratory animals of Xiamen University Laboratory Animal Center and is in compliance with the regulations on the management of laboratory animals of China and the Regulations on the Administration of Laboratory Animals of Fujian Province. Sixty male nude mice aged 6-7 weeks and weighing ~20-22 g, provided by a Shanghai Company, were used to establish IS models. All *in vivo* animal experiments were approved by the Ethical Committee for Animal Research at Xiamen University and carried out in accordance with the approved guidelines and regulations. The mice were placed in sterile cages (5 mice in each cage with sterilized corncob bedding) and ventilated in a pathogen-free room with a laminar flow hood, a 2 h light/12 h dark cycle, and fed with pressure cooker and water. Surgery in all animals was sterile during the experiment. Prior to dissection, mice were humanely euthanized by exposure to a rising concentration of CO_2_ gas.

### Photothrombosis stroke model

Herein, a photothrombosis method was used to establish an IS model in mice. Briefly, mice were made to inhale isoflurane into a deep anesthesia state and then the head was fixed with a positioner and the mice were placed on a feedback-controlled heating pad to maintain a body temperature of 37 ± 0.5 ℃ during the experimental period. The scalp was cut longitudinally (2.0-2.5 cm) to expose the skull of mice. A layer of membrane on the surface of the skull was gently scraped to make the coronal plane, vector midline, and anterior fontanelle clearly visible. The rose bengal solution (10 μL/g) was injected slowly through the tail vein. A laser beam at 532 nm with a diameter of 1.5 mm was irradiated ~2 mm on the outside of the anterior fontanelle for 15 min. The wound was then closed with a reverse cutting needle and nylon suture for imaging purposes.

### Middle cerebral artery occlusion (MCAO) stroke models

The preparation stage for the MCAO model was the same as that for the photothrombosis model mentioned above. After sterilizing the neck skin of mice in the supine position, the skin was opened with scissors to expose the common carotid artery. The sheath of the artery was carefully separated so as not to injure the vagus nerve. The external carotid artery and internal carotid artery were thus separated. The common and external carotid arteries were ligated with silk thread. The internal carotid artery was clamped with a clip and a small opening was cut with an ophthalmic scissor. A 6-0 silicone-coated monofilament was inserted into the common carotid artery, advancing toward the artery clip. The filament was secured in place with the distal silk tie and left in place for the duration of the occlusion.

### Hemorrhage transformation stroke models

The model of hemorrhagic transformed stroke was based on the MCAO model. After the MCAO model was successfully established, a 6-0 silicone-coated monofilament was inserted into the middle cerebral artery until the blood vessel was punctured, resulting in hemorrhagic stroke.

### PAI system

A hemispherical PAI system (Nexus 128 scanner, Endra Inc., Ann Arbor, MI) with an isotropic and homogeneous spatial resolution at 200 μm was employed to monitor the mouse brain. The laser wavelength was tunable from 680 to 950 nm for PA signal excitation by an optical parametric oscillator laser with a repetition rate of 20 Hz. PA signal detection was achieved through a hemispherical US device that consisted of 128 US transducers. The 128 US transducers (5 MHz central frequency) with a diameter of 3 mm were spirally positioned on a hemisphere with a curvature radius of 100 mm. The 128 PA signals were simultaneously triggered by a Q-switch trigger signal from the laser and recorded by a 128-channel high precision data acquisition system. The acquisition of a complete circular scan took ~1.7 min. A computer with a multi-core processor was equipped with a high-performance graphics processing unit for image reconstruction. The hemispherical PAI system adopted back-projection reconstruction algorithm as described in our previous work 40-42.

### PAI with co-registered US system

Mice were anesthetized using 2.5% isoflurane and secured onto a heated imaging platform underneath the PAI transducer. US gel was applied on top of the brain to facilitate US transmission. Experimental PAI with co-registered US was performed using a commercially available laser integrated high-frequency US system (Vevo^®^ LAZR, FujiFilm VisualSonics Inc.). The system consists of a tunable NIR Nd:YAG laser coupled into two fiber bundles attached tightly onto a 256-element linear array ultrasonic transducer on both sides. The laser signal synchronized the US signal acquisition.

PAI was performed using the following parameters: transducer, LZ-250; frequency, 21 MHz; depth, 20.00 mm; width, 23.04 mm; wavelength, 750/850 nm; gain, 18 dB for US and 43 dB for PAI; PA focal depth, 10 mm; acquisition mode, sO_2_/Hbt. Arrival time gain compensation was applied to account for PA signal loss with increased depth and kept constant for all imaging sessions. Three-dimensional PA images were acquired for the whole brain to estimate sO_2_ and hemoglobin concentration. After the mice were anesthetized, the ultrasonic transducer was placed above the brain of the mice. 3D scanning parameters were set according to the actual size of the nude mice brain with a step size of 0.08 mm and a scanning range of 18 mm.

Following imaging, animals were taken off and monitored to ensure full recovery. Post-processing of all imaging data was implemented using the Visualsonics^®^ workstation suite (VevoLab, ver1.7.2). PA-based estimates of sO_2_ and hemoglobin concentration were calculated using the two-wavelength approach as previously reported 43, 44. B-mode US was used to localize brain anatomy. Pseudo-color images representing sO_2_ were superimposed on spatially co-registered B-mode US gray images.

### PAM system

The brain microvessels of mice before and after IS were imaged by an optical resolution PAM system at 532 nm. In brief, after the mouse model was successfully established, the scalp of the mouse was opened by scissors. Then the brain area of the mouse was placed in the system for scanning.

The laser repetition rate of the system was 5 kHz with a pulse width of 7 ns. The acoustic signals generated by laser irradiation on the imaging tissues were amplified by a 50-dB amplifier (Mini-Circuits, USA) and then received by a 50 MHz ultrasonic sensor. The maximum amplitude projection images of the ROI were acquired by integrating the signals with the computer. The spatial resolution of the system was of 10 μm.

### *In vivo* MRI

After the IS model was established, the brain of mice was imaged by an MRI device before and six hours after modeling. *In vivo* T2-weighted MR images of mice were determined using a 9.4 T MR scanner (Biospec, Bruker) by using the multi-slice, multi-echo sequence with the following parameters: repetition time/echo time: 4000/9.5 ms; echo image: 10; slice thickness: 0.5 mm; field of view: 2 × 2 cm; matrix: 256 × 256. The normalized MR signal of IS was defined as the signal intensity of the scar area divided by that of the background.

### Histological analysis

Initially, mice were euthanized at the predetermined time points and were then perfused intracardially with 60 mL of 4% paraformaldehyde. The brains were removed and stored in 4% paraformaldehyde. The right brain damage areas were dissected and embedded in paraffin. A series of adjacent 5 μm horizontal sections of the brain injury region were cut and stained with H&E. On average, five slices per brain were prepared. Photographs were obtained at 200× magnification using an optical microscope equipped with a digital camera.

### Statistical analysis

Quantitative results were presented as mean ± standard deviation. Statistical differences among groups at different times were checked by one‐way repeated measures ANOVA with SPSS version 21 software (IBM Corp., Armonk, NY, USA). A probability value of *P <* 0.05 was considered statistically significant.

## Discussions

Timely diagnosis is essential for stroke patients. As a new non-invasive optical imaging modality, PAI has been widely used in pre-clinical research of various diseases, and indeed its use in the diagnosis of breast cancer is entering the stage of clinical trials 45-47. Based on previous studies, we used this technology to conduct more detailed and in-depth structural and functional imaging studies on IS.

In order to obtain more comprehensive, reliable, and rigorous results, we used two representative models of IS, and then verified the feasibility of this technology for the diagnosis of IS. PACT was able to locate the lesion directly and rapidly in the photochemistry IS model by EB dye within 5 min. The underlying mechanism is that when the BBB was intact, EB dye was unable to cross the BBB and accumulate in infarct areas. The partial destruction of the BBB occurred at the infarct site upon occurrence of IS. Thus, EB dye bound to the plasma albumin crossed the BBB and accumulated in the infarcted area of the cerebral cortex, significantly enhancing the PA signal. Similarly, in the MCAO model, cerebral cortical venous enlargement could be imaged by PACT even without EB dye, indirectly reflecting the pathological phenomenon around the infarction area at a very early stage. In general, PAI was able to directly locate the infarct area by EB dye in a photothrombosis model, and indirectly reflected the occurrence of disease through the physiological and pathological manifestations of the cerebral cortex after IS in the MCAO model. It was not possible to directly localize the infarct area in the MCAO model because (1) the infarct site in MCAO model mice is deeper than that of photothrombosis model mice, which is usually approximately 2 mm deep from the cerebral cortex, and (2) the absorption peak of EB dye is 610 nm, while the imaging wavelength is 680 nm, resulting in insufficient detection sensitivity. Most importantly, patients with IS often carry a considerably increased risk of hemorrhagic transformation, at which time thrombolytic therapy for patients will lead to a large probability of death. Our results indicated that PAI could directly reflect the risk of hemorrhagic transformation in patients with IS, thus guiding doctors to adopt a reasonable therapeutic regimen.

In terms of functional imaging, significant differences in sO_2_ levels between infarcted and normal areas could be observed by PACT, permitting effective functional parameters for the diagnosis of IS. Microscopically, PAM could be used to observe more subtle changes in vascular structure in the infarcted area and the related extent of BBB openness. These data were obtained by using three different types of PAI systems; by developing a system that combines the advantages of all three types, comprehensive information could be more conveniently retrieved at both the microscopic and macroscopic level and from structure to function.

Compared with conventional MRI, PAI can diagnose IS more quickly with higher resolution. Although PAI has the major advantage of offering greater depth imaging than traditional optical imaging, including two-photon or fluorescence imaging, deeper imaging still needs to be achieved. Currently, the laser energy of the PAI system used in the experiment is only 1.3 mJ/cm^2^; which is far below the safety threshold (40 mJ/cm^2^) of the American National Standards Institutes. We firmly believe that, with the development of this technology by using, for example, stronger laser energy, a higher sensitivity of US probe, and more advanced and efficient algorithms, deeper and more accurate PAI will be achieved in the near future.

## Conclusions

In conclusion, we used PACT to perform full-view structural and functional imaging of sO_2_ in IS at the macroscopic level, and then observed the microvascular changes in the infarcted area at the microscopic level by using PAM. Structurally, the results indicated that this technique could directly locate the infarct foci at unprecedentedly early stage (5 min after IS) in photothrombosis model. In MCAO model, cerebral cortical venous enlargement could be imaged by label-free PACT, indirectly reflecting the physiopathological features around the infarction area as soon as 3 min and also surveilling the obstruction of cerebral cortical venous return. In addition, the results also demonstrated that PACT can noninvasively detect hemorrhagic foci at very early stage (5 min). Functionally, there were significant changes of sO_2_ in infarcted areas in both models. The sO_2_ in the infarcted area decreased significantly compared with the normal area, which further indicated the occurrence of IS. These results represent a promising new perspective for IS diagnosis and also expand related pre-clinical study range as well as the disease progression mechanism study.

## Supplementary Material

Supplementary figures.

## Abbreviations

BBB: blood-brain barrier
EB: Evans blue
IS: Ischemic stroke
MCAO: middle cerebral artery occlusion
PA: Photoacoustic
PAI: Photoacoustic imaging
PACT: photoacoustic computed tomography
PAM: photoacoustic microscopy
ROI: region of interest
sO_2_: oxygen saturation
TTC: tetrazolium chloride
US: ultrasound
2D: two-dimensional
3D: three-dimensional
