# Supplementary material for: In vivo photoacoustic imaging dynamically monitors the structural and functional changes of ischemic stroke at a very early stage
Source: Theranostics. 2020 Jan 1;10(2):816–28. doi: 10.7150/thno.38554 (PMC6929999; doi:10.7150/thno.38554)
Supplement: Supplementary file 1 — Supplementary figures. [file thnov10p0816s1.pdf]

Supplemental material for

In vivo photoacoustic imaging dynamically monitors the structural and functional changes of ischemic stroke at a very early stage

Jing Lv‡, Shi Li‡, Jinde Zhang, Fei Duan, Zhiyou Wu, Ronghe Chen, Maomao Chen, Shanshan Huang, Haosong Ma, and Liming Nie*

State Key Laboratory of Molecular Vaccinology and Molecular Diagnosis & Center for Molecular Imaging and Translational Medicine, School of Public Health, Xiamen University, Xiamen, China

‡These authors contributed equally to this work.

Address: School of Public Health, Xiamen University, Xiang An Nan Road, Xiang An District, Xiamen 361102, Fujian Province, P. R. China

*Corresponding authors: Liming Nie, E-mail: nielm@xmu.edu.cn

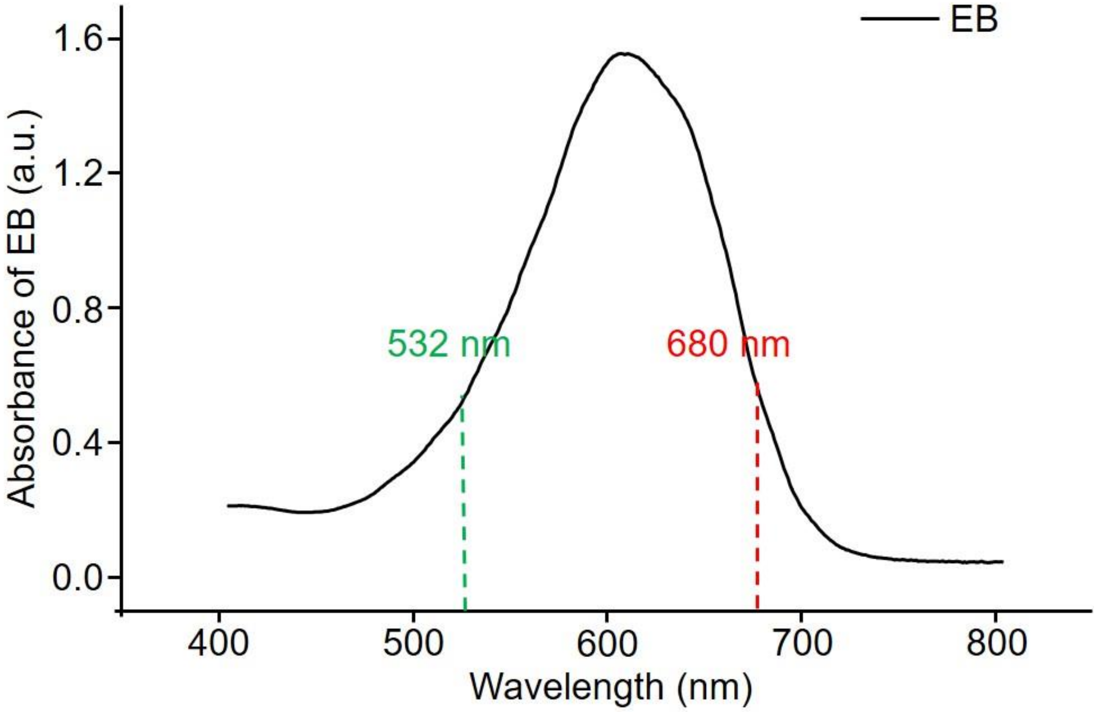

Figure S1 UV-Vis absorption curve of Evans blue (EB).

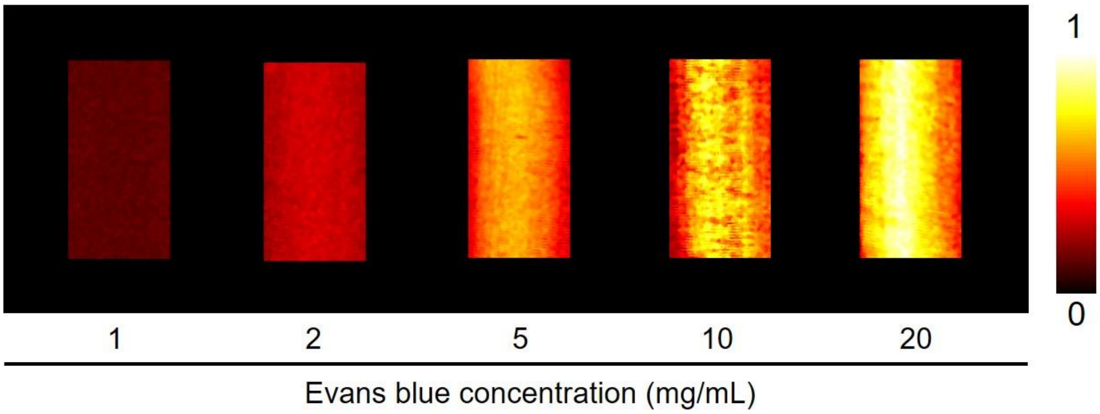

Figure S2 Photoacoustic microscopy (PAM) images of EB at different concentrations.

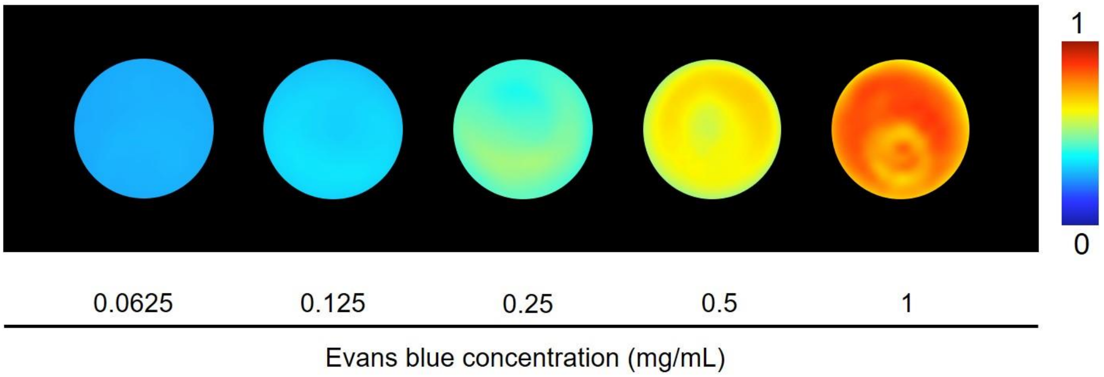

Figure S3 Photoacoustic computed tomography (PACT) of EB at different concentrations.

TTC

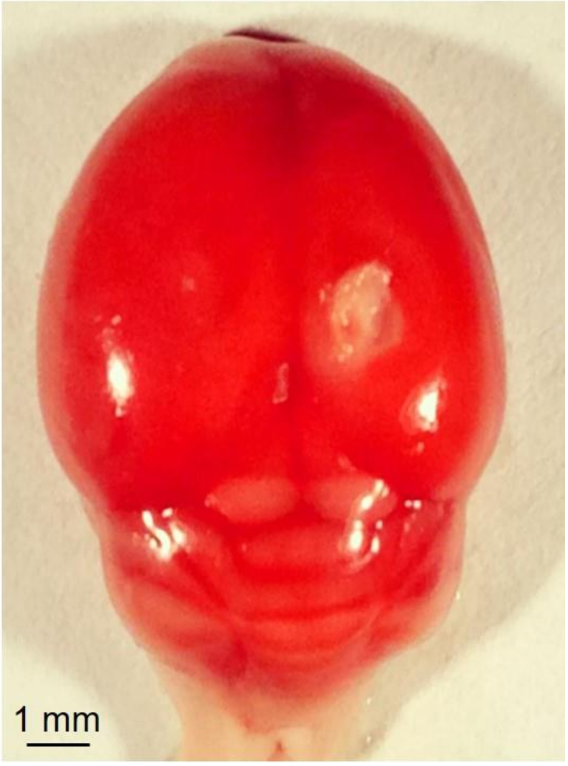

Figure S4 Triphenyl tetrazolium chloride (TTC) staining of a mouse brain in photothrombosis model.

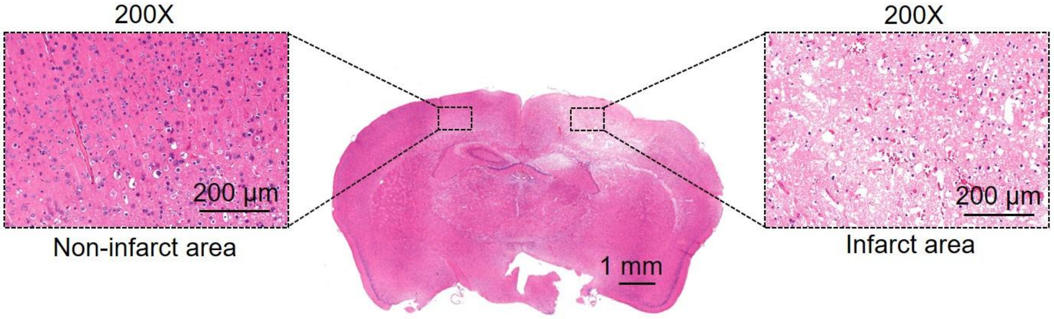

Figure S5 Hematoxylin and eosin (HE) staining (200-fold magnification) of a mouse brain after photothrombosis modeling in non-infarct and infarct areas, respectively.

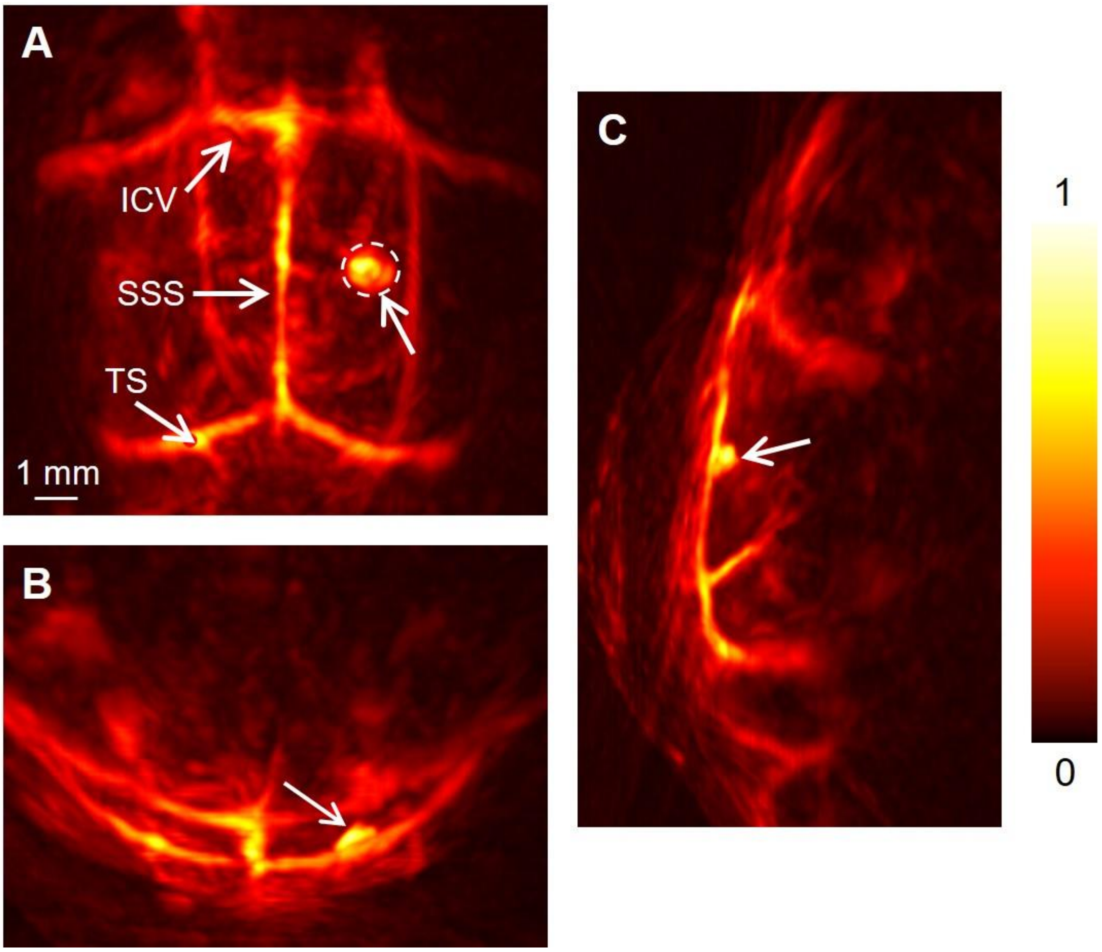

Figure S6 Triple-view PACT of a photothrombosis model mouse brain after EB dye injection at 680 nm. (**A**), (**B**), and (**C**) refer to coronal, transverse, and sagittal planes of the mouse brain, respectively.

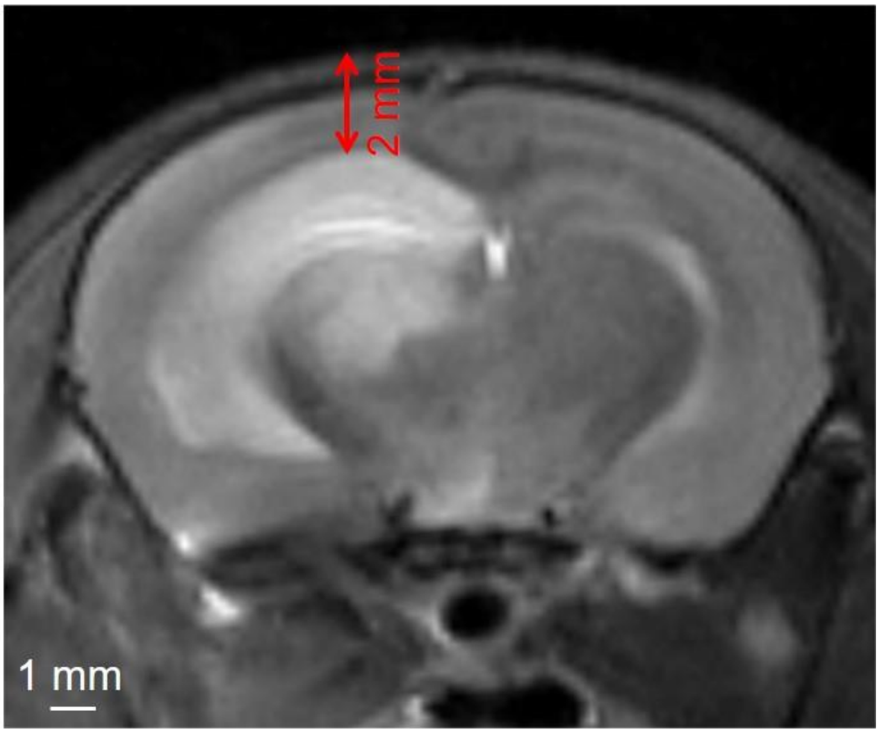

Figure S7 Magnetic resonance imaging (MRI) of an MCAO model mouse brain in coronal plane.

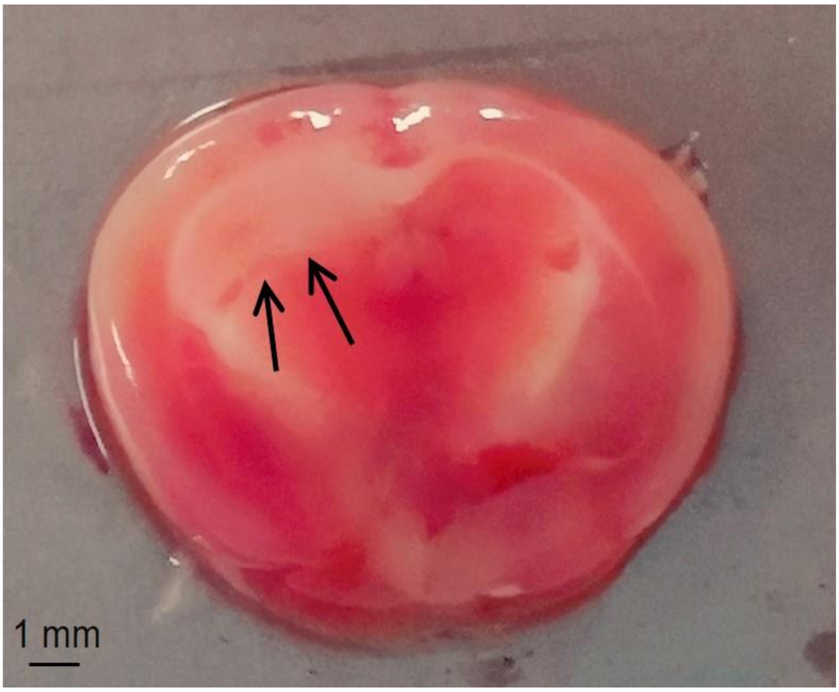

Figure S8 TTC staining of the brain of an MCAO model mouse.

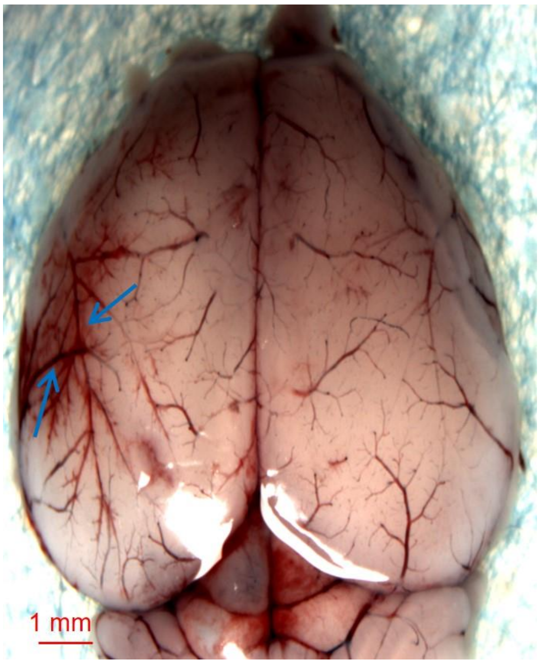

Figure S9 Photograph of a mouse brain in MCAO model. The arrows indicate blood vessels that were congested due to ischemic stroke.

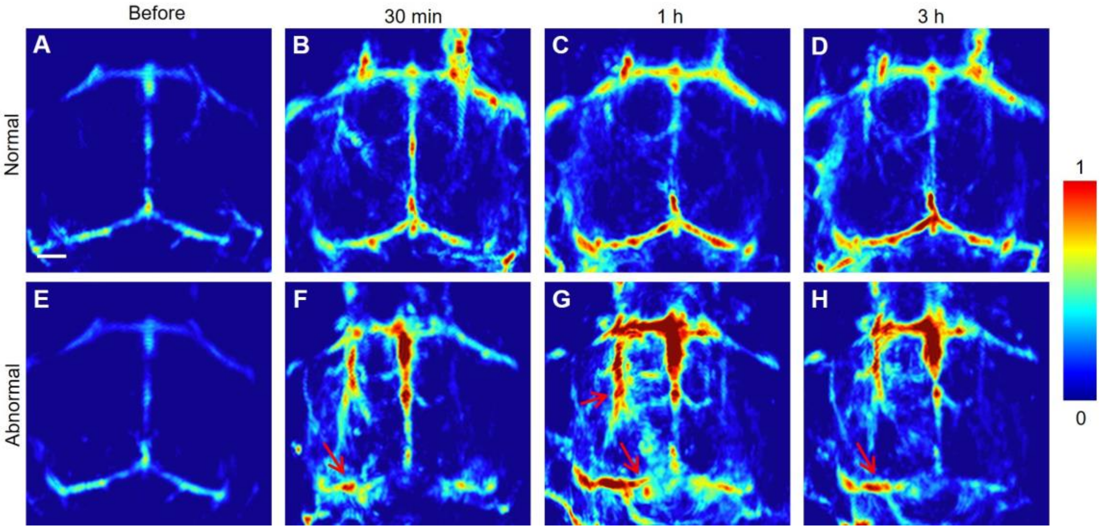

Figure S10 (**A–D**) Representative PA images of a normal mouse injected with EB at varied time points at 680 nm. (**E–H**) Representative PA images of an MCAO modeling mouse right followed by EB injection at varied time points at 680 nm.

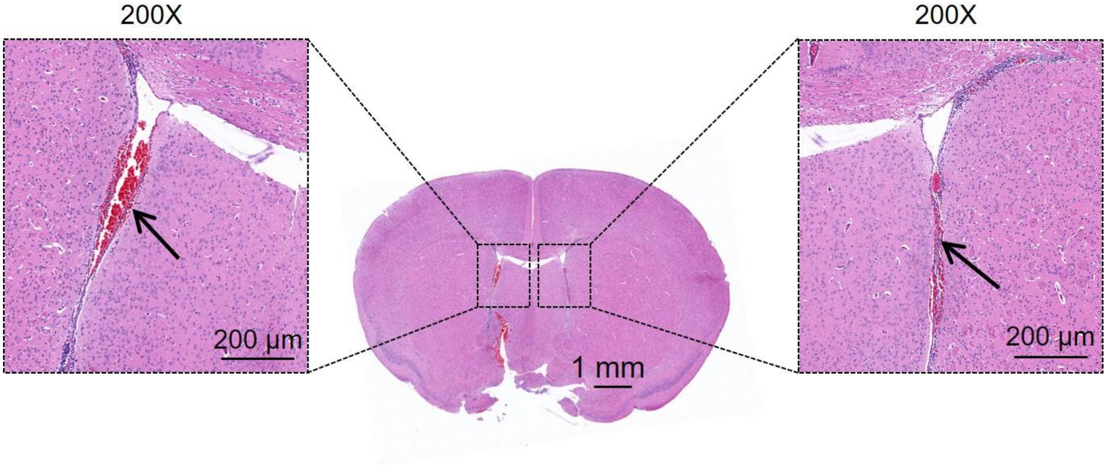

Figure S11 HE staining (200-fold magnification) of a mouse brain after hemorrhagic transformation modeling.

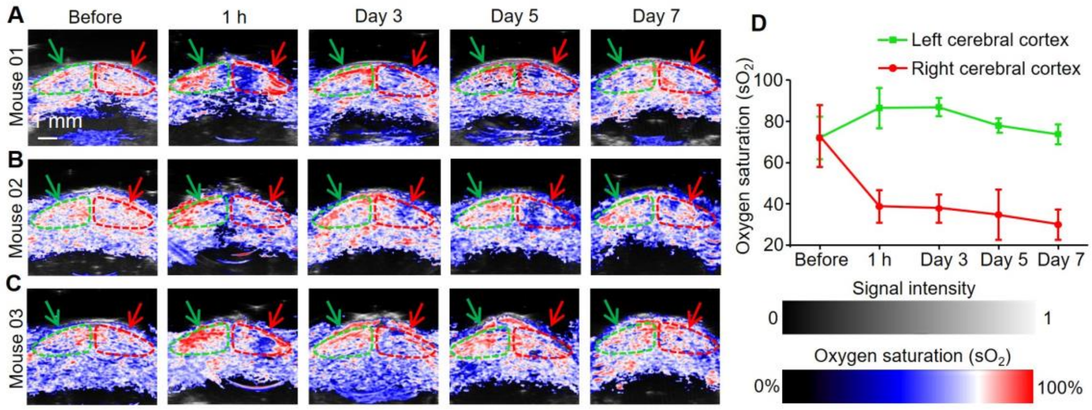

Figure S12 (**A–C**) Long-term photoacoustic functional images of brain oxygen saturation (sO_2) at different time points in three photothrombosis model mice. (**D**) Long-term statistical results of sO_2 mapping in apoplectic and normal areas at different time points of photothrombotic mice (n = 5; the error bars show the s.d.), respectively.
